# Hypertension and Associated Risk Factors Among the Sudanese Banking Sector in River Nile State: A Descriptive Cross-Sectional Study

**DOI:** 10.7759/cureus.24770

**Published:** 2022-05-06

**Authors:** Sufian M Khaild, Ziryab I Taha, Ommama I Ali, Mohammed H Mohammed, Yassein Abdelhai, Jimmy William

**Affiliations:** 1 Internal Medicine, Nile Valley University, Khartoum, SDN; 2 Internal Medicine, Haj El-Safi Teaching Hospital, Khartoum North, SDN; 3 Internal Medicine, University of Bahri, Khartoum, SDN; 4 Internal Medicine, Atbara Teaching Hospital, Atbara, SDN; 5 Transplant, Ahmed Gasim Hospital, Khartoum, SDN; 6 Internal Medicine, Sligo University Hospital, Sligo, IRL

**Keywords:** waist circumference, bmi, employees, banking sector, hypertension

## Abstract

Background

Hypertension is a global entity accounting for one of the most modifiable risk factors for all-cause morbidity and mortality. It is associated with a raised risk of cardiovascular disease, particularly in developing countries. Nevertheless, the banking sector profession lifestyle is sedentary and accompanied by high levels of mental stress, thus at a higher risk of developing hypertension.

Objective

The objective is to assess the prevalence of hypertension among bank employees and the associated risk factors in River Nile State - Sudan.

Methods

A descriptive cross-sectional study was performed on Atbara and Al-Damar localities' banks in River Nile state from January to March 2020. Data were collected on demographics, BMI, waist circumference (WC), medical history, family history, nutritional habits, physical activities, medications history, work stress, complaints, and blood pressure.

Results

Ninety-eight bank employees were enrolled, with elevated blood pressure present in 45(45.9%) participants, of whom 18 (40%) were newly diagnosed. 43.9% were in the age group 31-40 years. High blood pressure was significantly associated with older age >40 years, BMI > 30 kg/m^2^, WC > 90 cm, diabetes mellitus (DM), smoking, family history, salty diet, reduced daily exercise (30 minutes per day), severe stress at work, with overall P-value = <0.005.

Conclusion

The prevalence of high blood pressure was remarkably high among bank employees. Risk factors were: age (>40 years), obesity, DM, family history, salty diet intake, severe stress levels at work and sedentary lifestyle.

## Introduction

Hypertension (the silent killer) is the modern-day epidemic and becoming a significant medical and global public health issue due to its role in coronary heart disease, stroke, and other vascular complications [1]. Elevated blood pressure is a systolic blood pressure of ≥ 140 mmHg and diastolic blood pressure of ≥ 90 mmHg recorded in an individual [2]. The global prevalence of elevated blood pressure in adults aged 18 years and older was around 22% in 2014, with the number of individuals aged 30-79 years with hypertension doubling from 1990 to 2019 [3,4]. Approximately 9.4 million deaths and 7% of disease burden as computed by DALYs (disability-adjusted life years) resulted from raised blood pressure in 2010. [3] There is a direct correlation between increased blood pressure levels and the risk of stroke and coronary events, as it is directly responsible for 57% of all stroke deaths and 24% of all coronary heart disease deaths [5,6].

Hypertension is becoming a public health emergency worldwide, with the African region having the highest prevalence of high blood pressure worldwide at 27% in 2021 [7]. In addition, some studies projected an increase of 80% in the hypertensive population by 2025 [8]. Hypertension is a controllable disease, and a slight decline of 2 mmHg population-wide in BP can prevent 151,000 stroke cases. The prevalence of hypertension has increased by 30 times among the urban population over 55 years and about ten times among the rural population over 36 years [9].

Many modifiable factors contribute to the current high prevalence rates of hypertension. These include obesity, alcohol intake, physical inactivity, psychological stress, eating a salty diet, inadequate intake of fruits and vegetables, and socioeconomic determinants [3].

Hypertension is one of the most common self-reported occupational health challenges, with banking sector professions being at substantial risk given the sedentary working conditions and high levels of mental stress [6,10].

## Materials and methods

Study design

A descriptive cross-sectional community-based study.

Study area and duration

The study was conducted in banks in Atbara and Al-Damar localities between January 2020 and March 2020.

Study population

All banks' employees in Atbara and Al-Damar during the study period were involved.

Inclusion Criteria

All employees regardless of the job description and employees with a confirmed diagnosis of their comorbidities (cardiovascular diseases, diabetes mellitus [DM], metabolic syndrome, etc.) were included.

Exclusion Criteria

Refusal to participate. Patients who were newly discovered to have high blood pressure yet did not attend follow-up to confirm the diagnosis of Hypertension. Patients with an unconfirmed diagnosis of comorbidities.

Sample Size

The study enrolled 98 employees from nine banks during the whole study period.

Data collection tools and methods

An overview of the study goals was explained to all employees from all the banks initially. Those who agreed to participate have received a structured questionnaire comprising demographics, BMI, WC, job descriptions, medical history, family history, nutritional habits, physical activities, medications history, work stress, complaints and blood pressure. Data collection was carried out by two investigators, who ensured that all questionnaires were full filled out and helped the participants to fill them if required.

Measurements

BMI and WC

Height, weight, and waist circumference to assess BMI were measured using standardised equipment (stadiometer, electronic scales) and techniques by trained personnel. BMI was computed by dividing weight in kilograms by the square of their height in meters (kg/m^2^). Afterward, the BMI of each participant was categorised using WHO categories: underweight (<18.5 kg/m^2^), normal weight (18.5-24.9 kg/m^2^), overweight (25-29.9 kg/m^2^), class I obesity (30-34.9 kg/m^2^), class II obesity (35-39.9 kg/m^2^), and class III obesity (≥40 kg/m^2^) [11]. WC was measured with gentle breathing at the midpoint between the lowest rib and the iliac crest to the nearest 0.1 cm [12].

Blood Pressure

Blood pressure was recorded in both arms after resting for 10 minutes. It was made sure that caffeinated drinks or smoking were consumed half an hour before assessment as per JNC 7 guidelines [2].

It was measured three times, with the first measurement abandoned (to avoid possible anxiety). An average value of the second and third readings was taken for systolic and diastolic blood pressure.

Patients with high blood pressure with no previous history of hypertension have been followed up further before labelling as newly diagnosed hypertension.

As per the National Health Institute for Health and Care Excellence (NICE) guidelines [13], the blood pressure of participants was considered uncontrolled if it is above 140/90 in the age below 80 years and above 150/90 in those above 80 years.

Data analysis

Data were analysed using Statistical Package for Social Sciences (SPSS V. 21.0) software. The analysed data were presented in tables and figures designed by Microsoft Excel 2007. Chi-square test was used as a significance test, and the P-value was considered significant at level 0.05.

## Results

Overall, this study enrolled 98 bank workers, 72 (73.5%) were males, and 26 (26.5%) were females, the majority of which 43 (43.9%) were aged between 31 and 40 years. Considering job descriptions, 78 (79.6%) were cashiers, eight (8.2%) were security workers, seven (7.1%) were clerks, and five (5.1%) were managers, as shown in Table 1.

**Table 1:** Association between blood pressure and participants' demographic characteristics. J.D. means job description

Factor	Blood Pressure (mmHg)					
	Normal		130/90-150/100		>150/100	
	N	%	N	%	N	%
Age						
< 30	10	62.5	6	37.5	0	0
31 – 40	27	62.8	16	37.2	0	0
41 – 50	9	40.9	12	54.5	1	4.5
51 – 60	7	53.8	5	38.5	1	7.5
61 - 75	0	0	4	100	0	0
Gender						
Male	39	54.2	31	43.1	2	2.8
Female	14	53.8	12	46.2	0	0
J.D.						
Manager	0	0	5	100	0	0
Cashier	42	53.8	34	43.6	2	2.6
Clerk	4	57.1	3	42.9	0	0
Security	7	87.5	1	12.5	0	0

Elevated blood pressure was present in 45 (45.9%) employees, of which 43(43.9%) had blood pressure ranging from 130/90 to 150/100 mmHg and two (2%) above 150/100 mmHg. Nevertheless, 18 (40%) participants had undiagnosed hypertension and were followed up to confirm the diagnosis. Furthermore, most workers had elevated BMI (n=40; 40.8%), as well as waist circumference between 80 and 90 cm (n=34; 34.7%). However, only 20 (20%) of workers were smokers. Positive family history of hypertension was found in 16 (16.3%) workers, while hypertension combined with DM was found in 18 (18.4%) workers, with parents being more involved as family members (n=36; 36.7%) than siblings (n=10; 10.2%). Regarding nutritional history, most of the workers consumed a salty diet (n=70; 71.4%), with more than half adding extra salt during meals (n=52; 53.1%). Also, half of the participants (n=49; 50%) consumed fruits less than three times per week, and 61.2% (n=60) consumed vegetables daily and performed daily exercises for 30 minutes per day. Work stress spectrum was described as very severe, severe, moderate and minimal by 23 (23.5%), 42 (42.9%), 29 (29.6%), and four (4.1%) employees, respectively. More than 10% of the employees had symptoms associated with high blood pressure, chiefly headaches (n=37; 37.8%), blurring vision (n=15; 15.3%), and dizziness (n=10; 10.2%).

An extensive multivariate analysis, along with logistic regression showed that the following are significant independent determinants of elevated blood pressure among bankers; age group from 40 to 50 years (OR=2.6; 95%CI: 1.1-7.7; P-value= 0.000), age group from 51 to 60 years (OR=3.8; 95%CI: 1.5-9.3; P-value= 0.000), age group from 60 to 75 years (OR=6.5; 95%CI: 2.1-12.6; P-value= 0.000), BMI > 30 kg/m^2^ (OR=7.4; 95%CI: 3.8-16.6; P-value= 0.000), WC >100 cm (OR=6.5; 95%CI: 2.4-11.5; P-value= 0.000), DM (OR=3.7; 95%CI: 1.8-6.9; P-value= 0.036), smoking (OR=4.6; 95%CI: 1.9-9.7; P-value= 0.022), family history of heart disease (OR=7.5; 95%CI: 3.9-12.3; P-value= 0.000), family history of hypertension (OR=4.5; 95%CI: 2.7-10.1; P-value= 0.006), family history of DM (OR=3.2; 95%CI: 2-5.4; P-value= 0.011), family history of hypertension and DM (OR=2.8; 95%CI: 1.6-4.8; P-value= 0.04), salty foot intake (OR=3.1; 95%CI: 1.8-5.5; P-value= 0.021), add salt during eating (OR=3.3; 95%CI: 1.5-6.2; P-value= 0.018), 0.5 hour daily exercise (OR=2.8; 95%CI 1.4-7.9; P-value= 0.024), severe work stress (OR=3.4; 95%CI: 1.7-8.5; P-value= 0.004), very severe work stress (OR=6.5; 95%CI: 2.2-15.1; P-value= 0.000), headache (OR=2.4; 95%CI: 1.7-5.5; P-value= 0.038), dizziness (OR=2.1; 95%CI: 1.1-6.2; P-value= 0.027) and fatigue with nausea (OR=2.6; 95%CI: 1.4-4.6; P-value= 0.047) as shown in Table 2.

**Table 2:** The multivariate logistic regression showed determinants of elevated BP among bankers.

	Odds Ratio (OR)	95%CI	P-value
Age			
· 31-40	0.4	0.2-1.9	0.157
· 41-50	2.6	1.1-7.7	0
· 51-60	3.8	1.5-9.3	0
· 61-75	6.5	2.1-12.6	0
BMI			
· 25-30	1.4	0.89-3.3	0.175
· >30	7.4	3.8-16.6	0
Waist circumference (cm)			
· 80-90	0.18	0.03-1.03	0.648
· 91-100	0.31	0.06-1.7	0.181
· >100	6.5	2.4-11.5	0
Comorbidities			
· HTN+DM	7.2	3.6-19.4	0
· DM	3.7	1.8-6.9	0.036
· Other	1.1	0.2-2.4	0.519
Smoking (Yes)	4.6	1.9-9.7	0.022
Family history			
· Heart disease	7.5	3.9-12.3	0
· HTN	4.5	2.7-10.1	0.006
· DM	3.2	2.0-5.4	0.011
· HTN+DM	2.8	1.6-4.8	0.04
· Other	0.7	0.06-1.2	0.898
Salty food intake	3.1	1.8-5.5	0.021
Add salt during eating	3.3	1.5-6.2	0.018
Daily exercise rate			
· 0.5 hour/ day	2.8	1.4-7.9	0.024
· 0.5-1 hour/ day	0.4	0.09-1.3	0.243
Work stress			
· Moderate	0.8	0.3-2.2	0.705
· Severe	3.4	1.7-8.5	0.004
· Very severe	6.5	2.2-15.1	0
Headache	2.4	1.7-5.5	0.038
Dizziness	2.1	1.1-6.2	0.041
Blurring vision	3.6	1.2-6.6	0.027
Fatigue and nausea	2.6	1.4-4.6	0.047

## Discussion

To the best of our knowledge, this is the first study assessing the frequency and risk factors of hypertension among the banking sector profession in Sudan in general and in Atbara and Al-Damar localities, River Nile state particularly.

In the present study, elevated blood pressure was seen in 45.9% (n=45) of the employees; 18 (40%) were newly diagnosed. Similar to results in several studies from East-western regions of southern India and West Nigeria [10,14-16]; however, higher than results from studies in South Nigeria and Benin located in West Africa (12.4% and 25.3%, respectively) [17,18]. Nevertheless, higher outcomes were present in the east-western regions of North India (69.5% and 68%) [19,20]. These variations are attributed most likely to the differences in geographical areas, sample sizes, and blood pressure cut-off targets employed.

The ageing process is one of the main risk factors for developing hypertension, and the risk increases with age for both genders [21]. In this study, elevated blood pressure was significantly more common among employees above 40 years than their younger coworkers (P-value= 0.041). These findings are comparable with results from west Indian regions that reported a significant association between hypertension among bank employees aged 45 years and above [10,19]. Furthermore, the Framingham Heart Study concluded that the lifetime risk for people developing hypertension between 45 and 65 years of age is estimated to increase up to 90% [22].

Another risk factor for developing hypertension identified in our subjects was obesity, as high blood pressure was significantly associated with obese workers with BMI> 30 kg/m^2^ (OR: 7.4 [CI= 3.8-16.6], P-value= 0.0000 and those with WC above 100 cm (OR: 6.5 [CI: 2.4-11.5], P-value= 0. 000). Likewise, these findings correspond to Salaudeen et al. and Maroof et al., where hypertension among bankers was significantly associated with increased waist circumference and body mass index. In addition, a study by Imaad et al. reported that body mass index ≥ 25 kg/m^2^ and abnormal waist-hip ratio were significantly more frequent among the hypertensive than normotensive population [12]. Also, Singh et al. and Friedman et al. supported similar conclusions, with increasing BMI and WC being potential risk factors for hypertension [23,24].

Of note, the occurrence of high blood pressure was significantly associated with DM (P-value= 0.006). This has been previously observed by Ashwinkumar et al., Maroof et al., where hypertension among bankers was significantly associated with DM [19,20].

Smoking and tobacco use are linked to several mechanisms of elevated blood pressure levels [25,26]. This is no different from findings in our current study, where high blood pressure was significantly more frequent among smokers than non-smokers, with OR of 4.6 (P-value= 0.002). Similar results were seen in studies among bank workers reporting a significant association between smoking and hypertension [10,16].

Among our study participants, elevated blood pressure was significantly common in employees with positive family (P-value= 0.017), similar to studies from west India [10,15,16]. Many genes regulate blood pressure, but the exact mechanism is not yet fully understood. However, genes contribute to a greater extent than shared environment to the well-established familial correlation of blood pressure [27].

Furthermore, the present study demonstrated that elevated blood pressure was significantly associated with intake of salty foods (P-value= 0.0001), adding salt during eating (P-value= 0.008), which corresponds to data from India, reporting consumption of extra salt increased the occurrence of hypertension two-folds (OR=2.49) among bank employees [14].

Sedentary behaviour and lifestyle are associated with hypertension among the general population, and given that the banking profession necessitates extended hours of physical inactivity, they would be at higher risk of developing hypertension [28,29]. In our study, elevated blood pressure was significantly associated with low rates of daily exercise (P-value= 0.000), consistent with studies from India and Ethiopia emphasising the same entity [10,14,29].

Job-related stress has become a fundamental risk factor for hypertension, with evidence of a positive association between longer working hours and hypertension among workers [30]. In this study, severe and very severe working stressful statuses were significantly associated with elevated blood pressure (OR: 6.5 [2.2-15.1], P-value= 0.024). These findings were akin to Antoine et al. reporting that bank employees who described working under severe stress were eight times more likely to develop high blood pressure (OR=8, P-value=0.0027) [18].

Limitations

Our study has a few limitations. Firstly, the small sample size is mainly due to the lesser labour force in Sudanese banks compared to India (which hosts the highest number of publications on the same topic), given the difference in population size. Secondly, outcomes from this study cannot be generalised or applied to the local population as the banking sector employees are not representative of the general population regarding age, gender distribution, socioeconomic status, profession, and abode location.

Nevertheless, this current study enriches the existing literature with novel research emphasising the health issues of a workforce sector that is largely being overlooked. This issue is evident with the scarcity of publications from only Nigeria and India. We are in an era where primary prevention has a significant role in improving the overall morbidity and mortality; however, this improvement cannot be accomplished without underscoring the problem.

## Conclusions

The present study concluded that the frequency of high blood pressure was considerably high among bank employees in Al-Damar and Atbara localities in River Nile state, with a significant percentage being newly diagnosed. The risk factors of high blood pressure among banking sector professionals were age (>40 years), obesity and central obesity, DM, positive family history, salty diet intake, smoking, adding salt to meals, severe stress levels at work, and sedentary lifestyle.
